# Sub-noxious Intravesical Lipopolysaccharide Triggers Bladder Inflammation and Symptom Onset in A Transgenic Autoimmune Cystitis Model: A MAPP Network Animal Study

**DOI:** 10.1038/s41598-018-24833-x

**Published:** 2018-04-26

**Authors:** Paul Kogan, Suming Xu, Yaoqin Wang, Michael A. O’Donnell, Susan K. Lutgendorf, Catherine S. Bradley, Andrew Schrepf, Karl J. Kreder, Yi Luo

**Affiliations:** 1 Department of Urology, University of Iowa, Iowa City, IA USA; 2 Department of Psychology, University of Iowa, Iowa City, IA USA; 3 Department of Obstetrics and Gynecology, University of Iowa, Iowa City, IA USA

## Abstract

Patients with interstitial cystitis/bladder pain syndrome (IC/BPS) can potentially develop symptom flares after exposure to minor bladder irritants such as subclinical bacterial infection. To reproduce this symptom onset, we intravesically instilled a sub-noxious dose of uropathogenic *E. coli* component lipopolysaccharide (LPS) in young URO-OVA/OT-I mice, a transgenic autoimmune cystitis model that spontaneously develops bladder inflammation at ≥10 weeks of age. Female URO-OVA/OT-I mice (6-weeks old) were treated intravesically with phosphate-buffered saline (PBS) or PBS containing a sub-noxious dose (1 μg) of LPS. Mice were evaluated for bladder inflammation, pelvic pain, and voiding dysfunction at days 1, 7, and 14 post-treatment. Mice treated with LPS but not PBS developed early bladder inflammation with increased macrophage infiltration. Accordingly, the inflamed bladders expressed increased levels of mRNA for proinflammatory cytokines (IL-1β and IL-6) and pain mediator (substance P precursor). In addition, LPS-treated mice exhibited pelvic pain and voiding dysfunction such as increased urinary frequency and reduced bladder capacity. These functional changes sustained up to day 14 tested. Our results indicate that a single sub-noxious dose of intravesical LPS triggers early bladder inflammation and symptom onset in URO-OVA/OT-I mice, providing a useful model for IC/BPS symptom flare study.

## Introduction

Interstitial cystitis/bladder pain syndrome (IC/BPS) is a chronic inflammatory condition of the urinary bladder characterized by the hallmark symptoms of pelvic pain, urinary frequency and urgency^1^. During their lifespan, IC/BPS patients often experience symptom exacerbations (flares). These IC/BPS flares can vary in frequency, severity, and duration and severely affect patients’ quality of life^2^. Multiple factors can be a flare trigger including stress, sexual activity, diet, alcohol, and exercise, among others^2^. Urinary tract infection (UTI) has also been noted to be a potential flare trigger in IC/BPS patients^2–4^. While the actual incidence of significant bacteriuria was low based on standard urine culture^3^, sequencing analysis of DNA extracted from patients’ urine revealed the presence of numerous bacterial species^5^. These observations suggest that subclinical bacterial infection can be a potential risk factor for bladder inflammation and symptom flares in IC/BPS patients.

The etiology of IC/BPS remains elusive but has been associated with various conditions including autoimmunity. Patients with IC/BPS have been observed to develop antinuclear and anti-urothelium autoantibodies^6^, overexpress HLA-DR molecules on the urothelium^7^, and coexist with some autoimmune disorders such as systemic lupus erythematosus, Sjogren’s syndrome, and rheumatoid arthritis^1,6,8,9^. Immunosuppressive drugs have also been used to treat IC/BPS and demonstrated to be beneficial for some patients^10,11^. Moreover, bladder histology data have revealed a role of cell-mediated inflammatory mechanisms in IC/BPS^12,13^. Hence, autoimmune inflammation is likely a component of the pathophysiology of IC/BPS in subgroups of patients.

Animal models with experimental autoimmune cystitis (EAC) have been actively used in IC/BPS research since the 1990s^14–20^. These EAC models can be developed through immunization with bladder tissue components in rodents and used to reproduce many clinical correlates of human IC/BPS such as cystitis pain and voiding dysfunction^18–20^. We previously used genetic engineering technology to develop a transgenic EAC model (URO-OVA) that expresses the membrane form of the model antigen ovalbumin (OVA) as a self-antigen on the urothelium^21^. URO-OVA mice develop bladder inflammation at day 7 after adoptive transfer of OVA-specific CD8^+^ T cells from OT-I mice^21–25^ and resemble many clinical features of human IC/BPS including pelvic pain, voiding dysfunction, and increased mast cell counts^21,24,26^. Subsequently, we crossed URO-OVA mice with OT-I mice to generate URO-OVA/OT-I mice that spontaneously develop bladder inflammation at ≥10 weeks of age^21,23^. In this study, we used URO-OVA/OT-I mice to investigate whether intravesical administration of a sub-noxious dose of uropathogenic *Escherichia coli* (*E*. *coli*) component lipopolysaccharide (LPS) triggers early bladder inflammation and symptom onset in the animal model.

LPS is a cell wall component of Gram-negative bacteria including uropathogenic *E. coli* and acts as a virulence factor (endotoxin) to elicit strong inflammatory responses during bacterial infection^27^. Intravesical administration of LPS has been demonstrated to induce bladder inflammation characterized by edema and leukocytic infiltration in mice^28,29^, which was associated with increased expression of neuro-inflammatory factors in the bladder such as tumor necrosis factor (TNF)-α, substance P (SP), and nerve growth factor (NGF)^30,31^. These neuro-inflammatory factors are thought to play important roles in mediating cystitis pain^30,31^. Prior studies also demonstrated that intravesical LPS-induced bladder inflammation had the similar features of edema and leukocytic infiltration in rats, which was associated with increased expression of macrophage migration inhibitory factor (MIF) in the bladder^32^. Other inflammatory factors such as the bradykinin-1 receptor (a bradykinin receptor), neurokinin-1 receptor (an SP receptor), and nuclear factor-kappa B (a nuclear transcription factor) were also observed to involve in LPS-induced bladder inflammation and functional changes^33–35^. LPS is commonly used at a dose of 15 µg for intravesical induction of bladder inflammation in C57BL/6 mice^29,31,35^. We previously observed no induction of bladder inflammation in C57BL/6 mice after intravesical instillation of LPS at a dose of 1 µg^36^. Here we report that this low dose of intravesical LPS triggers early bladder inflammation and symptom onset in URO-OVA/OT-I mice (the same C57BL/6 genetic background), mimicking symptom flares seen in IC/BPS patients.

## Results

### URO-OVA/OT-I mice develop early bladder inflammation upon intravesical instillation of a single sub-noxious dose of LPS

**Figure 1:**
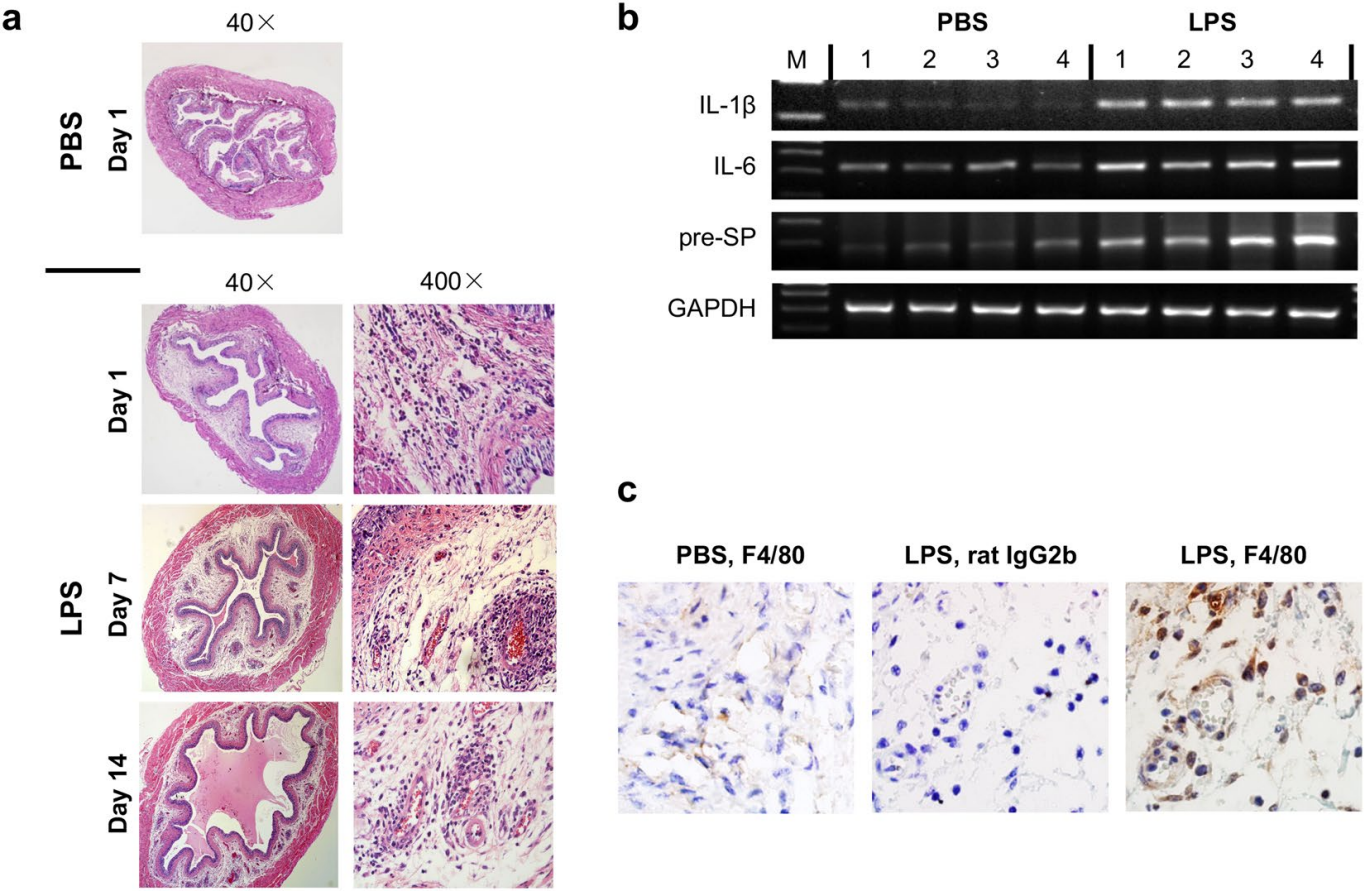
URO-OVA/OT-I mice develop early bladder inflammation upon intravesical instillation of a single sub-noxious dose of LPS. (**a**) URO-OVA/OT-I mice develop early bladder inflammation. The bladders of 6-week-old URO-OVA/OT-I mice were instilled with 100 µl PBS or 1 µg of LPS in 100 µl PBS. At days 1, 7 and 14 post-treatment with LPS the bladders were collected and processed for histological H&E staining. PBS-treated bladders were collected and processed for histological analysis at day 1 post-treatment. Magnification: x40 and x400. The images are representative of 11 PBS-treated bladders and 12, 8 and 8 LPS-treated bladders for day 1, 7 and 14, respectively. (**b**) The inflamed bladders of URO-OVA/OT-I mice express increased inflammatory factor mRNAs after LPS treatment. The bladder total RNAs were extracted 24 hours after intravesical PBS or LPS treatment and analyzed by RT-PCR for IL-1ß, IL-6, and substance P precursor (pre-SP) mRNAs. GAPDH was used as an internal control. Four bladders for each group are presented. M: a 100 bp DNA ladder. This figure panel is cropped from 4 different gels run and exposed in the same experimental conditions. Their corresponding full-length gels are presented in Supplementary Figures 1–4. (**c**) The inflamed bladders of URO-OVA/OT-I mice show increased macrophage infiltration after LPS treatment. The bladder immunohistochemistry was performed 24 hours after intravesical PBS or LPS treatment. *Left panel*, the bladder of a mouse treated with intravesical PBS and stained with a rat anti-mouse F4/80 antibody (IgG2b); *Middle panel*, the bladder of a mouse treated with intravesical LPS and stained with a control rat IgG2b; *Right panel*, the bladder of a mouse treated with intravesical LPS and stained with a rat anti-mouse F4/80 antibody (IgG2b). The images are representative of 4–5 mice for each group. Magnification: x1,000.

During the normal life history, URO-OVA/OT-I mice do not develop bladder inflammation until ≥10 weeks of age^21,23^. However, compared to the control 6-week-old URO-OVA/OT-I mice treated with intravesical phosphate buffered saline (PBS) (n = 11; score: - for all mice), 10 of 12 of sex- and age-matched URO-OVA/OT-I mice developed clear bladder inflammation (n = 12; score: - for 2, +for 2, ++for 2, and +++ for 6) 24 hours after intravesical instillation of a single sub-noxious dose of LPS (1 μg of LPS) (Fig. 1a, Table 1). The bladder inflammation sustained up to day 14 tested, although the intensity of bladder inflammation slightly reduced at the late timepoint (day 7: n = 8, score: - for 0, + for 1, ++ for 2, and +++ for 5; day 14: n = 8, score: - for 0, + for 2, ++ for 3, and +++ for 3) (Fig. 1a, Table 1). The LPS-treated bladders exhibited interstitial edema, mucosal hyperemia, and cellular infiltration in the lamina propria. In parallel with bladder histopathology, the inflamed bladders (day 1) expressed elevated levels of mRNAs for IL-1β, IL-6, and substance P precursor (pre-SP) as detected by RT-PCR (Fig. 1b) and increased F4/80 positive cell (macrophage) infiltration as detected by immunohistochemistry (Fig. 1c). These observations indicate that the bladders of URO-OVA/OT-I mice are highly sensitive to minor irritants and readily develop early inflammation upon stimulation with a single sub-noxious dose of LPS.

**Table 1:** Bladder response to a single sub-noxious dose of intravesical LPS in URO-OVA/OT-I mice.

	Bladder Inflammatory Score			
	−	**+**	**++**	**+++**
PBS Day 1 (n = 11)	11	0	0	0
LPS Day 1 (n = 12)	2	2	2	6
LPS Day 7 (n = 8)	0	1	2	5
LPS Day 14 (n = 8)	0	2	3	3

### LPS-induced early bladder inflammation is associated with pelvic pain in URO-OVA/OT-I mice

**Figure 2:**
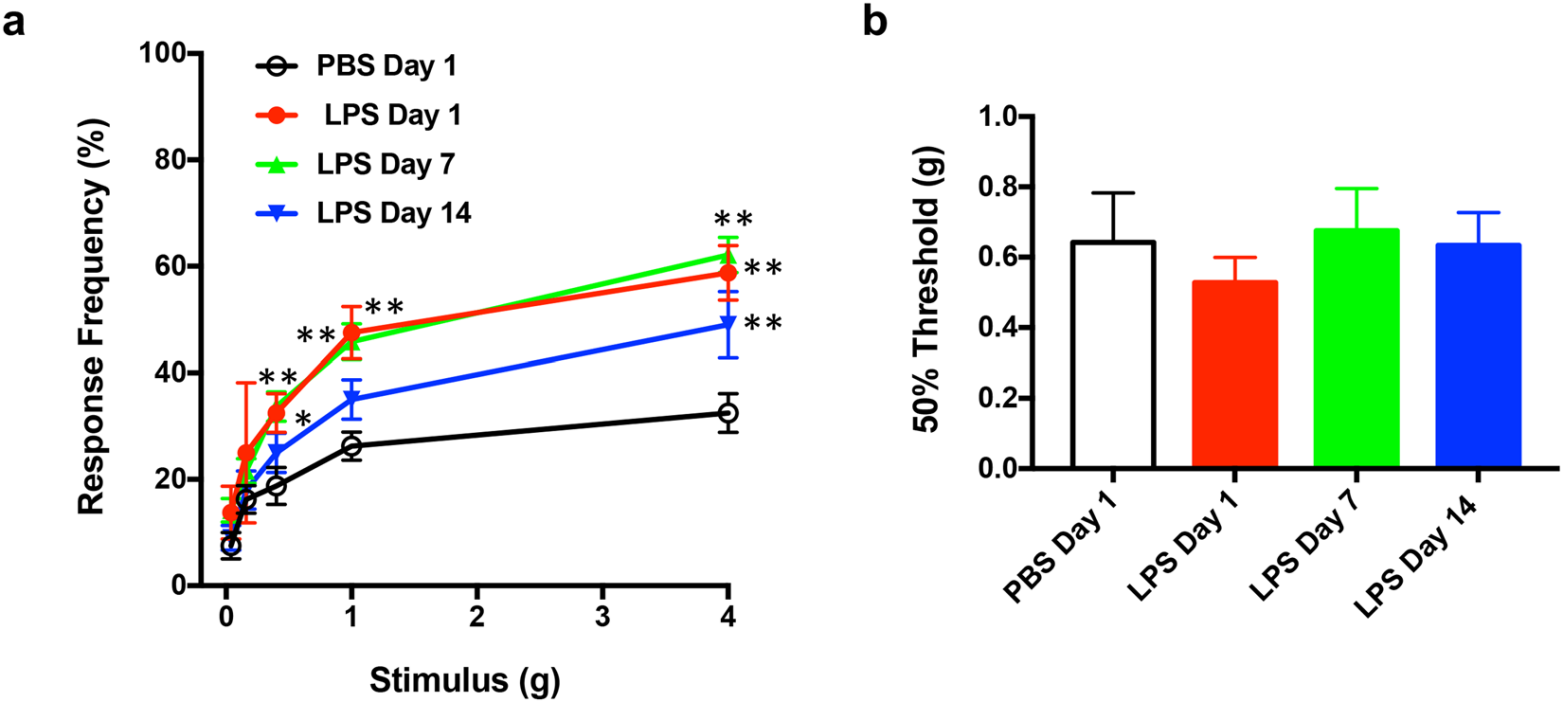
LPS-induced early bladder inflammation is associated with pelvic pain in URO-OVA/OT-I mice. (**a**) Six-week-old URO-OVA/OT-I mice were treated intravesically with 1 µg of LPS in 100 µl PBS and evaluated for pelvic response to von Frey filament stimulation at days 1 (n = 8), 7 (n = 19) and 14 (n = 10) post-treatment. Sex- and age-matched URO-OVA/OT-I mice treated with intravesical PBS at day 1 post-treatment (n = 8) were included for comparison. Data are shown as mean ± SEM percent of response frequency. *p < 0.05 and **p < 0.01 as compared to the PBS-treated group. (**b**) The same LPS-treated URO-OVA/OT-I mice exhibited no significant changes in tactile sensitivity (50% threshold) of the plantar region of the hind paw at 1, 7, and 14 days as compared to the PBS-treated URO-OVA/OT-I mice.

Six-week-old URO-OVA/OT-I mice were intravesically instilled with a single sub-noxious dose of LPS (1 μg of LPS) and evaluated for pelvic pain using von Frey filament stimulation at days 1, 7, and 14 post-treatment (Fig. 2a, Supplementary Table S1). Sex- and age-matched URO-OVA/OT-I mice treated with intravesical PBS at day 1 post-treatment were included for comparison. Compared to PBS-treated mice (n = 8), LPS-treated mice exhibited significantly increased pelvic response at day 1 (n = 8; 0.4 g: 32.5 ± 3.66 vs 18.75 ± 3.504, *p* < 0.05; 1 g: 47.5 ± 4.91 vs 26.25 ± 2.631, *p* < 0.01; 4 g: 58.75 ± 5.154 vs 32.5 ± 3.66, *p* < 0.01). This increased pelvic response sustained at day 7 (n = 19; 0.4 g: 33.684 ± 2.779, *p* < 0.01; 1 g: 45.79 ± 3.361, *p* < 0.01; 4 g: 62.105 ± 3.296, *p* < 0.01) and up to day 14 (n = 10; 4 g: 49.0 ± 6.227, *p* < 0.01). There were no significant differences in tactile sensitivity (50% threshold) of the plantar region of the hind paw between PBS- and LPS-treated groups (Fig. 2b; *p* = 0.871 by ANOVA). These observations indicate that URO-OVA/OT-I mice readily develop pelvic-restricted pain upon intravesical stimulation with a single sub-noxious dose of LPS in this experimental setting.

### LPS-induced early bladder inflammation is associated with voiding dysfunction in URO-OVA/OT-I mice

**Figure 3:**
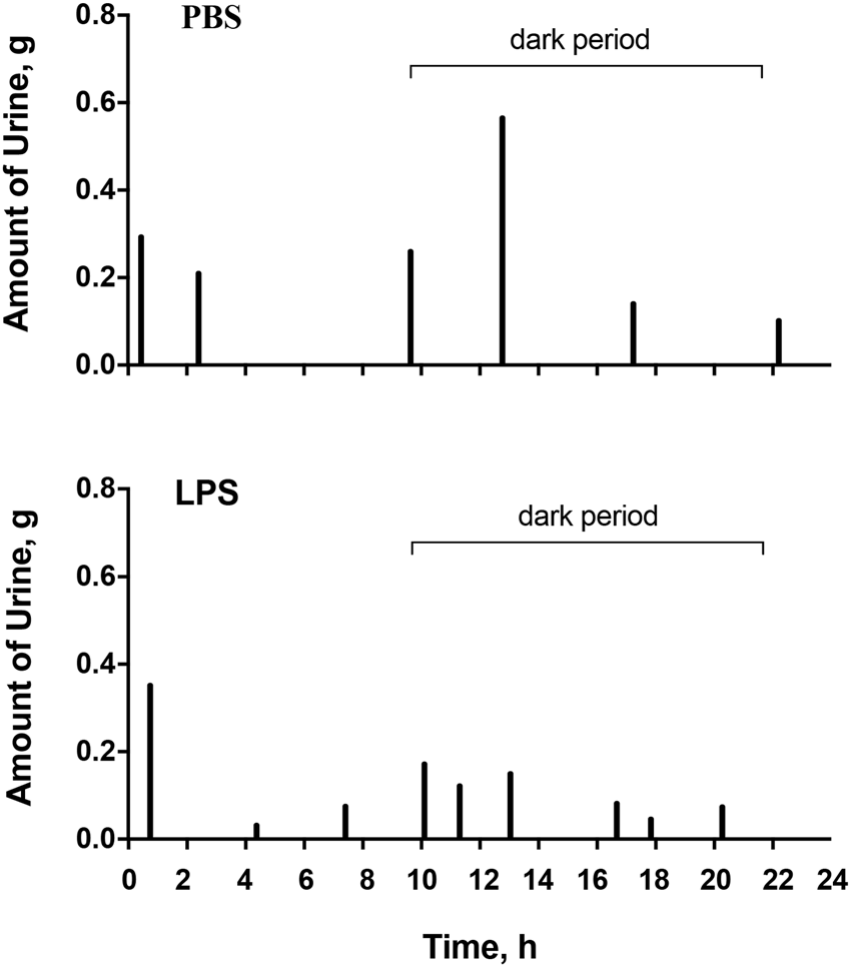
LPS-induced early bladder inflammation is associated with voiding dysfunction in URO-OVA/OT-I mice. Six-week-old URO-OVA/OT-I mice were treated intravesically with 100 μl PBS (*top panel*) or 1 µg of LPS in 100 µl PBS (*bottom panel*). After 24 hours, mice were evaluated for voiding habits using micturition cages. The results are representative of 5 PBS- and 7 LPS-treated mice.

Six-week-old URO-OVA/OT-I mice were intravesically instilled with a single sub-noxious dose of LPS (1 μg of LPS) and evaluated for voiding habits using micturition cages at days 1, 7, and 14 post-treatment. Sex- and age-matched URO-OVA/OT-I mice treated with intravesical PBS at day 1 post-treatment were included for comparison. There were no significant changes in voiding habits after a single intravesical PBS treatment compared to baseline voiding habits in the animal model (Supplementary Table S2). Compared to PBS-treated mice (n = 5), LPS-treated mice exhibited significant changes in voiding habits at day 1 post-treatment (n = 7) (Fig. 3, Table 2). These changes included decreased average volume voided per micturition (0.154 ± 0.012 vs 0.28 ± 0.033, *p* < 0.0001), decreased maximum volume voided per micturition (0.337 ± 0.054 vs 0.56 ± 0.048, *p* = 0.004), and increased total number of voids (9.429 ± 0.571 vs 5.2 ± 0.583, *p* < 0.0001). The number of voids in both light and dark periods was also significantly increased (in light: 3.571 ± 0.571 vs 2.0 ± 0.316, *p* = 0.019; in dark: 5.857 ± 0.404 vs 3.2 ± 0.49, *p* = 0.01). This voiding dysfunction sustained at days 7 (n = 10) and 14 (n = 5) (Table 2), although the numbers of voids in the light and dark periods were not statistically significant between the groups at these timepoints. The total voided volumes in 24 hours were similar between PBS- and LPS-treated groups (PBS day 1: 1.467 ± 0.254 g; LPS day 1: 1.471 ± 0.203 g; LPS day 7: 1.203 ± 0.152 g; LPS day 14: 1.224 ± 0.214 g; *p* = 0.6383 by ANOVA). These observations indicate that URO-OVA/OT-I mice readily develop voiding dysfunction upon intravesical stimulation with a single sub-noxious dose of LPS.

**Table 2:** Changes in voiding habits after a single sub-noxious dose of intravesical LPS in URO**-**OVA/OT-I mice (*p*-value: One-way ANOVA with LSD post test).

	PBS Day 1 (n = 5)	LPS Day 1 (n = 7)	LPS Day 7 (n = 10)	LPS Day 14 (n = 5)	*p*-value
Average volume voided per micturition, g	0.28 ± 0.033	0.15 ± 0.012	0.17 ± 0.015	0.17 ± 0.019	0.0008
Maximum volume voided per micturition, g	0.56 ± 0.048	0.34 ± 0.054	0.32 ± 0.037	0.29 ± 0.043	0.0044
Total number of voids	5.2 ± 0.583	9.43 ± 0.571	7.3 ± 0.578	7.4 ± 0.748	0.0023
in light	2.0 ± 0.316	3.57 ± 0.571	2.7 ± 0.260	3.0 ± 0.447	0.1087
in dark	3.2 ± 0.490	5.86 ± 0.404	4.4 ± 0.541	4.4 ± 1.030	0.0645

## Discussion

IC/BPS is one of the most refractory diseases in urology today, and the effort to develop animal models that can reproduce the clinical correlates of the human disease is greatly needed for therapeutic development. Since the etiology of IC/BPS remains elusive and many factors can be causative for the disease, animal models with diverse pathological pathways have been developed^26,37,38^. Due to their presence of OVA “self” antigen on the urothelium and endogenous OVA-specific CD8^+^ T cells in the immune system, URO-OVA/OT-I mice spontaneously develop bladder inflammation at ≥10 weeks of age^21,23^. Although these mice show no bladder inflammation prior to 10 weeks of age, the autoimmune condition renders the mice hypersensitive to otherwise sub-noxious bladder irritative stimuli such as a low dose of LPS. We have observed that URO-OVA/OT-I mice exhibit a low threshold trigger for producing exaggerated responses to a single sub-noxious dose of LPS. Intravesical instillation of LPS at 1 µg efficiently induced early bladder inflammation, pelvic pain, and voiding dysfunction in the animal model. This low dose of LPS used for cystitis induction in URO-OVA/OT-I mice was only 1/15 dose commonly used for cystitis induction in C57BL/6 mice^29,31,35^. This feature of the URO-OVA/OT-I model is clinically relevant, as a subclinical bacterial infection is suggested to be a potential risk factor for symptom flares in some IC/BPS patients^2–4^.

We recently reported that the same single sub-noxious dose of intravesical LPS induced acute bladder inflammation in a monocyte chemoattractant protein (MCP)-1 expressing transgenic cystitis model (URO-MCP-1)^36^. The bladder inflammation developed in URO-MCP-1 mice was also associated with pelvic pain and voiding dysfunction seen in IC/BPS patients^36^. Despite its constitutive expression of MCP-1 by the urothelium, the URO-MCP-1 model does not spontaneously develop bladder inflammation in the unmanipulated state. However, like the URO-OVA/OT-I model, the URO-MCP-1 model develops bladder phenotypic and functional changes upon intravesical instillation of 1 µg LPS^36^. Our observations indicate that a single sub-noxious dose of intravesical LPS is capable of triggering bladder inflammation and symptom onset in both IC/BPS-like animal models, supporting the hypothesis that a subclinical bacterial infection can be causative for bladder inflammation and symptom flares in human IC/BPS.

The URO-OVA/OT-I model develops early bladder inflammation, pelvic pain, and voiding dysfunction such as increased urinary frequency and reduced bladder capacity following intravesical instillation of a single sub-noxious dose of LPS. Although it is evident that a single sub-noxious dose of intravesical LPS triggers early bladder inflammation and symptom onset in the URO-OVA/OT-I model, the mechanisms by which LPS triggers bladder phenotypic and functional changes have not been identified. We have observed that the inflamed bladders of URO-OVA/OT-I mice expressed increased levels of CD8 and interferon (IFN)-γ mRNAs, suggesting that activation of endogenous OVA-specific CD8^+^ T cells may play an important role in LPS-induced early bladder inflammation and symptom onset in the animal model. In addition, the increased expressions of IL-1ß, IL-6 and pre-SP mRNAs in the inflamed bladders suggested that multiple inflammatory pathways, such as the Toll-like receptor (TLR), inflammasome and SP pathways, gained activation and contributed to the phenotypic and functional changes in the animal model. We plan to explore the mechanisms underlying the bladder phenotypic and functional changes in the animal model.

In this study we used intravesical *E. coli* LPS to mimic bladder infection by uropathogenic *E. coli*. LPS is the major component of the outer membrane of Gram-negative bacteria including *E. coli*. and acts as an endotoxin to elicit strong inflammatory responses during bacterial infection^27^. A study revealed that *E. coli* LPS inoculation was equally potent to, if not stronger than, *E. coli* inoculation for the induction of IL-6, nitric oxide (NO), and inducible NO synthase (iNOS) expressions in mouse bladders^39^. In addition, protein kinase C (PKC) activation and detrusor contraction were similarly increased in the bladders of mice treated with intravesical *E. coli* LPS or intravesical *E. coli*^39^. Other studies revealed that, similar to *E. coli* bladder infection^40,41^, intravesical *E. coli* LPS induced bladder pain and voiding dysfunction in mice^40,42^. These observations indicate that intravesical *E. coli* LPS can reproduce many features of *E. coli* bladder infection. However, it should be noted that *E. coli* LPS inoculation is not equivalent to *E. coli* inoculation, as *E. coli* LPS inoculation cannot reproduce bacterial colonization and associated pathological changes seen in *E. coli* inoculation.

The limitations of this study include the gender restriction of the URO-OVA/OT-I model, as only female mice were used due to a higher incidence of IC/BPS in females than males in humans^1,43^. The other limitations of this study include the lack of direct evaluation of bladder nociception as well as the lack of analysis of bladder inflammatory factors at the protein levels. In addition to exploring the mechanisms of the URO-OVA/OT-I model, our future studies will include the use of this animal model for therapeutic development, as URO-OVA/OT-I mice are responsive to anti-inflammatory agents such as dimethyl sulfoxide (DMSO)^23^, the only agent approved for intravesical treatment of IC/BPS by the Food and Drug Administration (FDA).

In summary, our results indicate that a single sub-noxious dose of intravesical LPS triggers early bladder inflammation and symptom onset in URO-OVA/OT-I mice, providing a useful model for IC/BPS symptom flare study.

## Materials and Methods

### Ethics statement

All animal procedures were approved by University of Iowa Animal Care and Use Committee (Permit Number: 1308153) and performed in accordance with the Guide for the Care and Use of Laboratory Animals of the National Institutes of Health.

### Mice

URO-OVA/OT-I mice were generated through crossbreeding of URO-OVA mice, a transgenic EAC model previously developed in our laboratory^21^, with OT-I mice (Jackson Laboratories, Bar Harbor, ME), a transgenic line that expresses CD8^+^ T cell receptor specific for the H2-K^b^/OVA_257–264_ epitope^25^. Both URO-OVA and OT-I mice are of C57BL/6 genetic background. URO-OVA/OT-I mice (F1 generation) retain the urothelial OVA expression and endogenous OVA-specific CD8^+^ T cells and develop spontaneous bladder inflammation at ≥10 weeks of age^21,23^. All mice were housed in a pathogen-free facility at the University of Iowa Animal Care Facility. Female mice were used due to a higher incidence of IC/BPS in females than males in humans^1,43^.

### Cystitis induction

Female URO-OVA/OT-1 mice (6 weeks old) were anesthetized through intraperitoneal (IP) injection of a mixture solution of ketamine (87.5 mg/kg) and xylazine (12.5 mg/kg). The bladder was then catheterized via the urethra with a 24 gauge, 3/4′′long plastic intravenous catheter (Smiths Medical, Southington, CT), instilled with 1 µg of LPS (*E. coli* 055:B5, Sigma-Aldrich, St. Louis, MO) in 100 µl PBS, and retained for 1 hour. Control mice were instilled with 100 µl PBS alone in the bladders.

### Bladder histology

Bladders were collected and processed for standard histological hematoxylin and eosin (H&E) staining as described previously^21^. Also, bladder inflammation was scored in a blinded manner based on infiltration of inflammatory cells in the lamina propria and the presence of interstitial edema as described previously^21^.

### Bladder immunohistochemistry

Bladder immunohistochemistry was performed as described previously^36^. Biotinylated rat anti-mouse F4/80 antibody (BioLegend, San Diego, CA; clone: CI:A3-1; rat IgG2b) was used to detect macrophages in the bladder tissue sections, while biotinylated rat IgG2b (BioLegend, San Diego, CA; clone: RTK4530;) was used as a control. Slides were developed using streptavidin-horseradish peroxidase complex (SAv-HRP) and diaminobenzidine (DAB) substrate solution (BD PharMingen), counterstained with hematoxylin solution, and photographed using an Olympus BX-51 microscope.

### Pelvic pain analysis

Pelvic pain was evaluated using 5 selected von Frey filaments (Stoelting Co., Wood Dale, IL) and presented as the percentage of positive response to each filament as described previously^36^. Tactile sensitivity of the plantar region of the hind paw was also evaluated using the same von Frey filaments and presented as the 50% withdrawal threshold to the filament stimulation as described previously^36^.

### Voiding habit analysis

Voiding habits were evaluated using micturition cages (Columbus Instruments, Columbus, OH) as described previously^24,36^. Urinary frequency, voided volume per micturition, and total urine volume were recorded and analyzed using Oxymax software (Columbus Instruments, Columbus, OH) as described previously^24,36^.

### Reverse transcriptase-polymerase chain reaction (RT-PCR)

As described previously^24,36^, total RNAs were extracted from the bladder using Qiagen RNeasy Mini Kit (Qiagen, Valencia, CA), followed by cDNA synthesis using Invitrogen SuperScript III Reverse Transcriptase (Invitrogen, Carlsbad, CA) and Oligo dT. PCR amplification was then performed on cDNA products using Taq DNA polymerase (New England Biolabs, Ipswich, MA) and sequence-specific primer pairs: 5′-GCCCATCCTCTGTGACTCAT-3′ and 5′-AGGCCACAGGTATTTTGTCG-3′ (230 bp) for IL-1ß, 5′-GTTCTCTGGGAAATCGTGGA-3′ and 5′-GGAAATTGGGGTAGGAAGGA-3′ (339 bp) for IL-6, 5′-GCCAATGCAGAACTACGAAA-3′ and 5′-GCTTGGACAGCTCCTTCATC-3′ (280 bp) for tachykinin-1 (substance P precursor), and 5′-GTTCCAGTATGACTCCACT-3′ and 5′-GTGCAGGATGCATTGCTG-3′ (321 bp) for glyceraldehyde-3-phosphate dehydrogenase (GAPDH). The housekeeping gene GAPDH was amplified for 25 cycles, while other molecules were amplified for 40 cycles. The PCR products were run on 1% agarose gels, stained with ethidium bromide, and imaged by Gel Doc EZ Imager (Bio-Rad Laboratories, Hercules, CA).

### Statistical analysis

Data were analyzed using Statistics Package for Social Sciences (SPSS 13.0, Chicago, IL), presented as mean ± SEM for both pelvic pain and voiding habit changes, and compared by Student’s *t*-test (two groups) or ANOVA followed by LSD post hoc tests (multiple groups). A value of *p* < 0.05 was considered statistically significant.

### Data Availability Statement

All data are fully available without restriction.

## Electronic supplementary material

Supporting Information
