# Supplementary material for: Sub-noxious Intravesical Lipopolysaccharide Triggers Bladder Inflammation and Symptom Onset in A Transgenic Autoimmune Cystitis Model: A MAPP Network Animal Study
Source: Sci Rep. 2018 Apr 26;8:6573. doi: 10.1038/s41598-018-24833-x (PMC5919907; doi:10.1038/s41598-018-24833-x)

Supporting Information

Title of the manuscript: Sub-noxious Intravesical Lipopolysaccharide Triggers Bladder Inflammation and Symptom Onset in A Transgenic Autoimmune Cystitis Model: A MAPP Network Animal Study

The list of authors: Paul Kogan, Suming Xu, Yaoqin Wang, Michael A. O'Donnell, Susan K. Lutgendorf, Catherine S. Bradley, Andrew Schrepf, Karl J. Kreder, Yi Luo

Supplementary Table S1. Changes in pelvic response to von Frey filament stimulation after a single sub-noxious dose of intravesical LPS in URO-OVA/OT-I mice (*p*-value: One-way ANOVA analysis with LSD post test. $^{*}p<0.05$ and $^{**}p<0.01$ compared to PBS-treated group [Student's t test]).

	Pelvic Stimulation Force				
	**0.04 g**	**0.16 g**	**0.4 g**	**1 g**	**4 g**
**PBS Day 1 (n=8)**	7.5±2.5	16.25±2.631	18.75±3.504	26.25±2.631	32.5±3.66
**LPS Day 1 (n=8)**	13.75±4.978	25.0±13.177	32.5±3.660*	47.5±4.910**	58.75±5.154**
**LPS Day 7 (n=19)**	14.21±2.205	21.58±2.327	33.68±2.779**	45.79±3.361**	62.11±3.296**
**LPS Day 14 (n=10)**	9.0±2.333	18.0±3.590	25.0±3.727	35.0±3.727	49.0±6.227**
***p*-value**	0.291	0.355	0.016	0.002	0.001

Supplementary Table S2. Voiding habits in URO-OVA/OT-I mice – baseline versus intravesical PBS treatment. There were no significant changes in voiding habits after a single intravesical PBS treatment compared to baseline voiding habits in the animal model (*24 hours after intravesical PBS treatment. *p* value: compared to baseline group [Student's *t* test]).

	**Baseline (n=7)**	**PBS* (n=7)**	***p*-value**
**Average volume voided per micturition, g**	0.281 ± 0.0245	0.303 ± 0.030	0.579
**Maximum volume voided per micturition, g**	0.493 ± 0.043	0.498 ± 0.050	0.943
**Total number of voids**	5.0 ± 0.436	4.143 ± 0.404	0.175
in light	1.857 ± 0.261	2.0 ± 0.309	0.730
in dark	3.286 ± 0.286	2.143 ± 0.459	0.056
**Total volume of voids, g**	1.371 ± 0.134	1.201 ± 0.106	0.340

Supplementary Figure S1. A full-length gel for RT-PCR analysis of IL-1β mRNA expressed by the bladders of URO-OVA/OT-I mice.

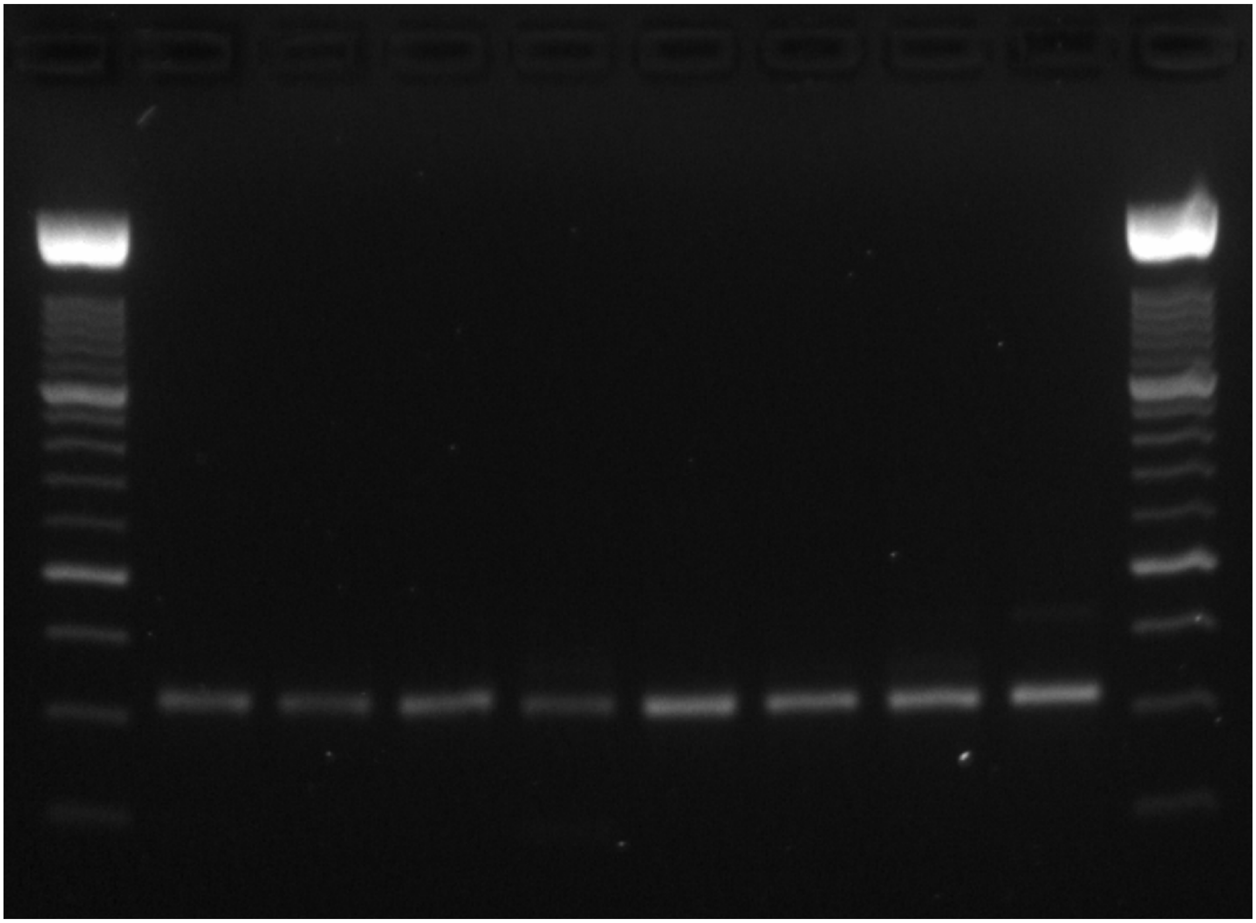

Supplementary Figure S2. A full-length gel for RT-PCR analysis of IL-6 mRNA expressed by the bladders of URO-OVA/OT-I mice.

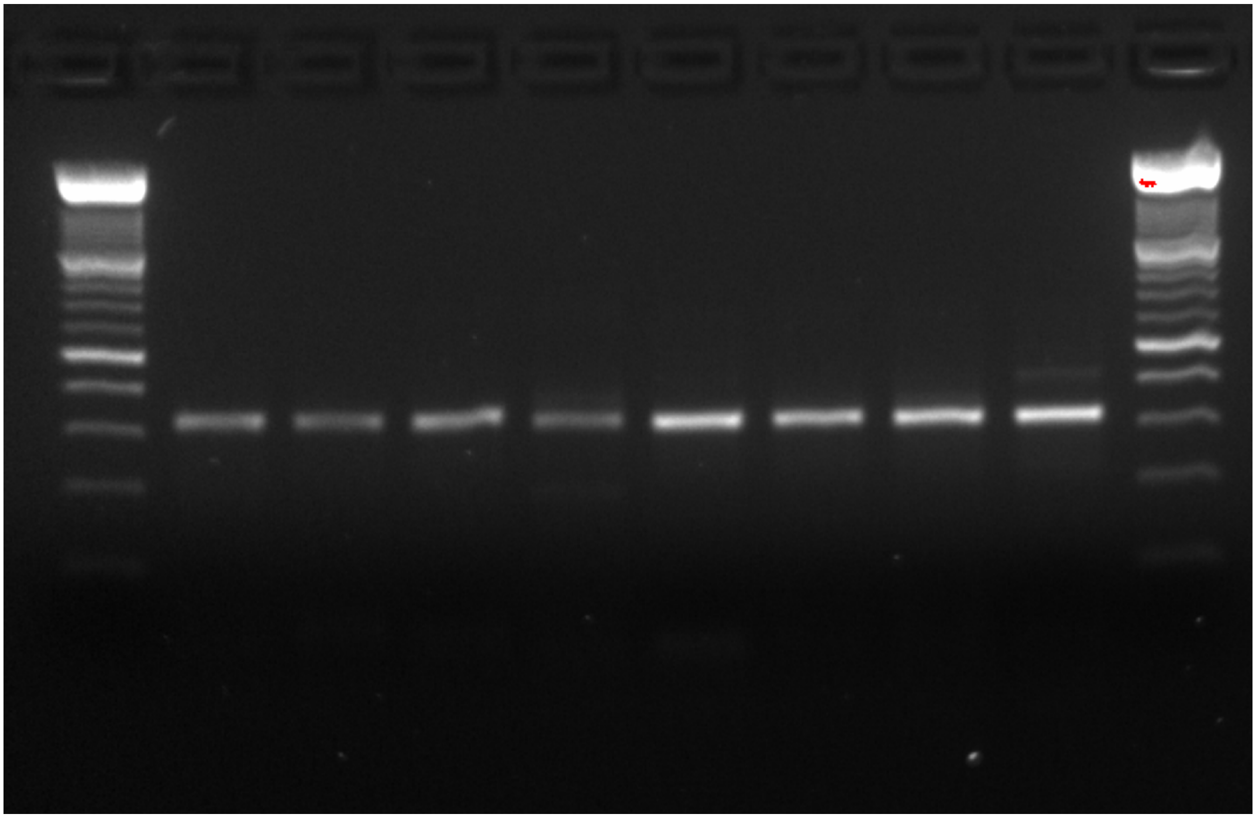

Supplementary Figure S3. A full-length gel for RT-PCR analysis of pre-SP mRNA expressed by the bladders of URO-OVA/OT-I mice.

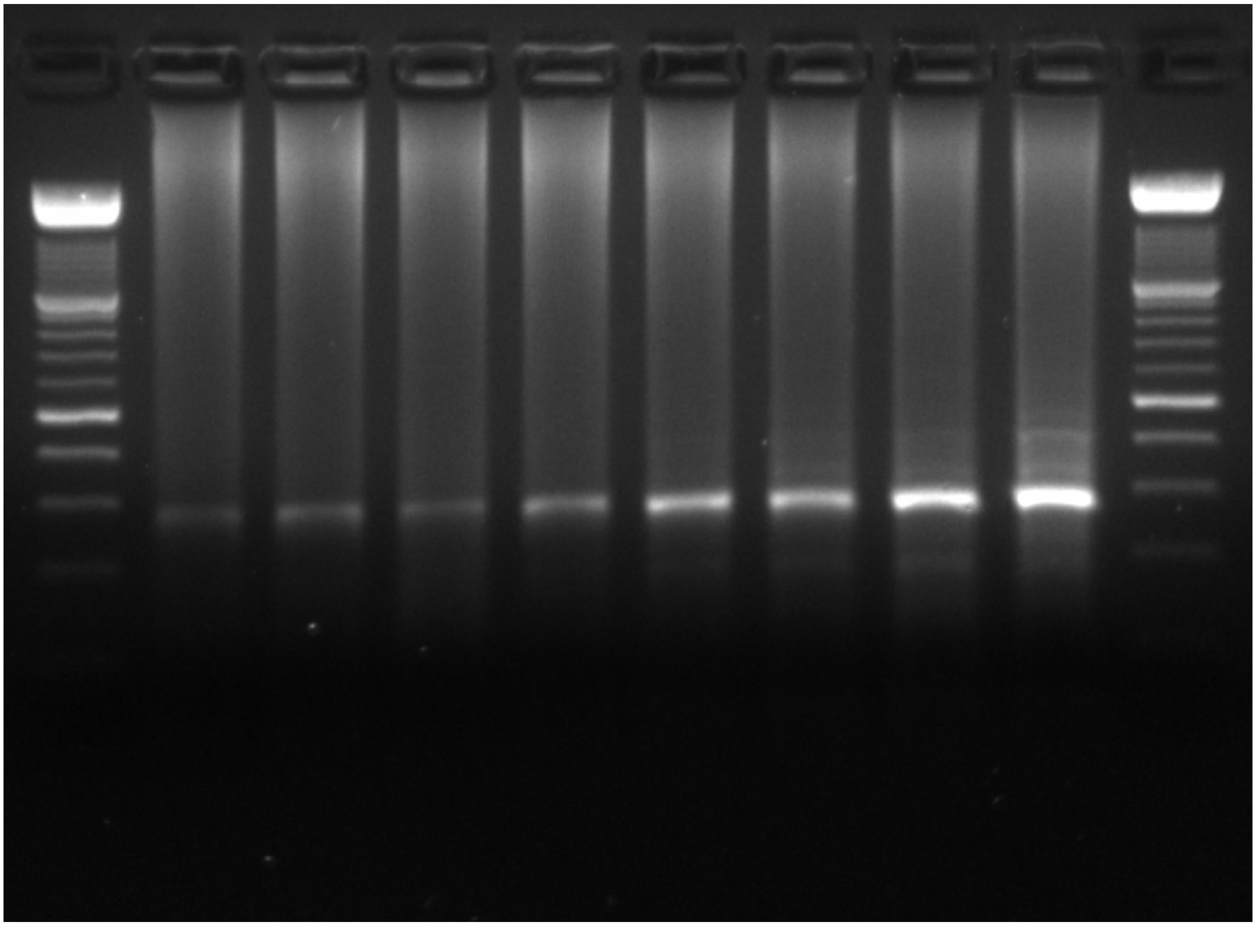

Supplementary Figure S4. A full-length gel for RT-PCR analysis of GAPDH mRNA expressed by the bladders of URO-OVA/OT-I mice.

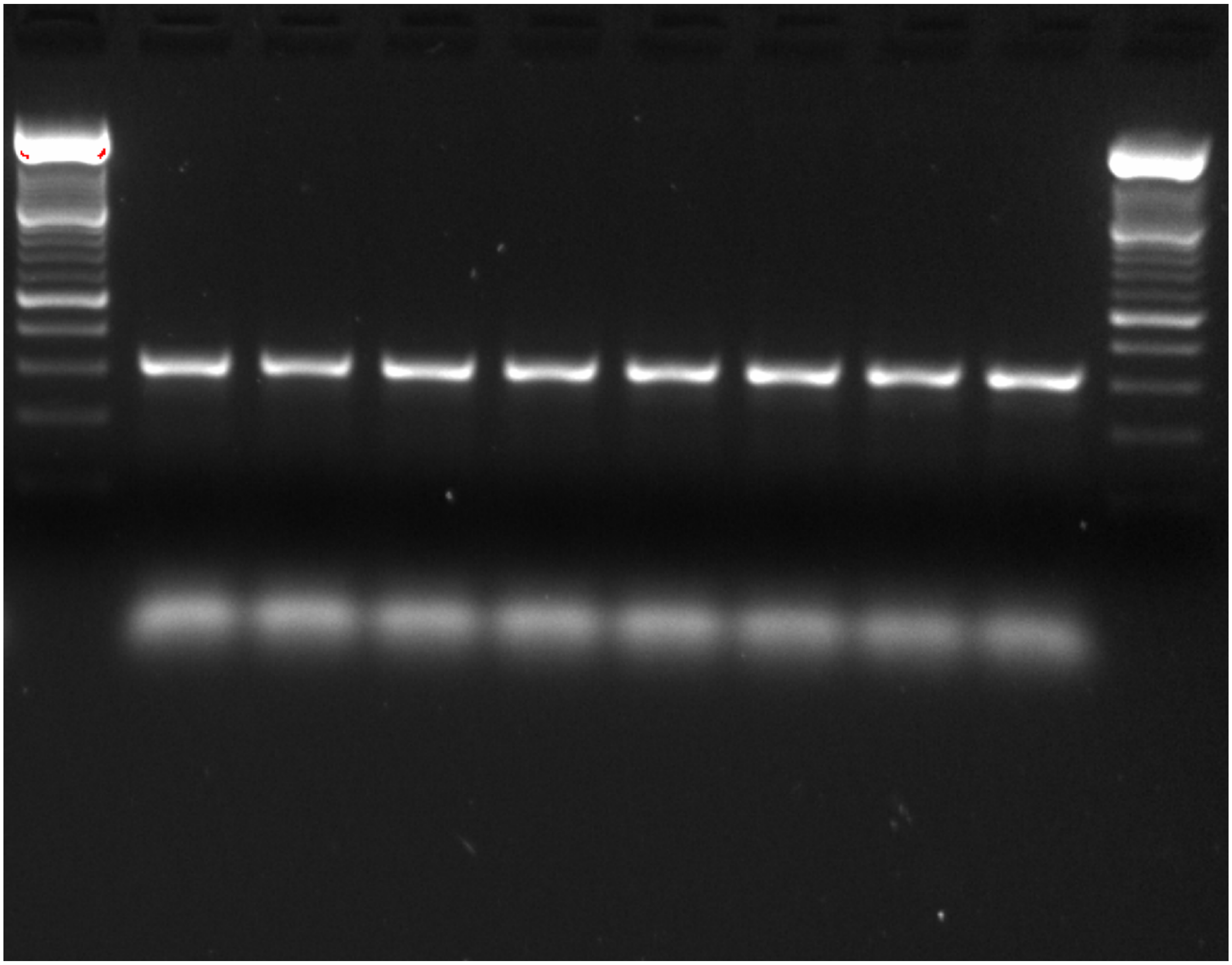

Supplement: Supplementary file 1 — Supporting Information [file 41598_2018_24833_MOESM1_ESM.pdf]
